# RSV vaccine development: advances and fusion protein-focused strategies

**DOI:** 10.3389/fimmu.2026.1710895

**Published:** 2026-02-25

**Authors:** Wang Xu, Revansiddha H. Katte, Maolin Lu

**Affiliations:** Department of Cellular and Molecular Biology, School of Medicine, The University of Texas at Tyler Health Science Center, Tyler, TX, United States

**Keywords:** fusion protein, monoclonal antibodies, prophylaxis, respiratory syncytial virus, RSV, vaccines

## Abstract

Respiratory syncytial virus (RSV) is a leading cause of acute lower respiratory tract infections, particularly affecting infants, young children, older adults, and immunocompromised individuals. While RSV infection often causes mild, cold-like symptoms, it can progress to severe pulmonary disease in these vulnerable populations, frequently necessitating hospitalization. Decades of research have defined the RSV fusion protein, particularly its prefusion form, as the primary target for prophylaxis and vaccine design. This has advanced transformative milestones, including the approval of long-acting monoclonal antibodies (Nirsevimab, Clesrovimab) and the vaccines for adults (Arexvy, Abrysvo, mRESVIA). This review begins with RSV molecular virology, summarizes the evolution of prophylactic antibody, and revisits lessons from past vaccine failures. It then emphasizes the current landscape of RSV vaccine development, categorizing platforms such as mRNA, protein subunit, virus-like particle/nanoparticle, live-attenuated, and viral vector approaches, highlighting both licensed vaccines and leading candidates under clinical evaluation. This review overviews current RSV vaccines and antibody prophylaxis and aims to inform the development of future prevention strategies.

## 1 Introduction

Respiratory syncytial virus (RSV) is one of the most significant pathogens causing respiratory infections in infants, young children, older adults, and immunocompromised populations, contributing to a substantial global disease burden (1, 2). In infants, RSV is a leading cause of bronchiolitis and pneumonia (3). According to the World Health Organization (WHO), RSV infections result in approximately 100,000 deaths annually among children under five years of age, around 3 million hospitalizations, and approximately 33 million cases of lower respiratory tract infections (LRTI) worldwide (1). Most of these cases occur in low- and middle-income countries, and neither the incidence nor mortality rates have declined over time (4).

**Figure 1:**
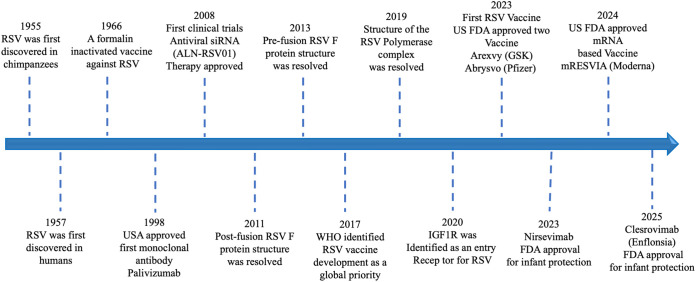
Timeline of key milestones in RSV research and vaccine development. Major milestones in RSV research and vaccine development are summarized. RSV was first discovered in chimpanzees (1955) and subsequently in humans (1957). Early efforts included a formalin-inactivated RSV vaccine (1966), which failed due to vaccine-associated enhanced disease. The first monoclonal antibody for prophylaxis, palivizumab, was approved in the United States (1998). Antiviral approaches were explored, including clinical trials of the siRNA therapy ALN-RSV01 (2008). Structural biology advances resolved the post-fusion (2011) and prefusion (2013) F protein conformations, the latter becoming a key target for modern vaccine design. In 2017, the World Health Organization designated RSV vaccine development as a global priority. Further progress included resolution of the RSV polymerase complex structure (2019) and identification of IGF1R as a viral entry receptor (2020). In 2023, the U.S. FDA approved the first RSV vaccines, Arexvy (GSK) and Abrysvo (Pfizer), for older adults, as well as the long-acting monoclonal antibody Nirsevimab for infant protection. In 2024, Moderna’s mRESVIA became the first mRNA-based RSV vaccine to be approved. Most recently, in 2025, the FDA approved another monoclonal antibody, Clesrovinmab (Enflonsia), for infant protection.

Decades of research in molecular virology and structural biology have accelerated the development of prophylactic antibodies and vaccines, providing more options for preventing RSV infection. Some antibodies and vaccines have progressed to widespread clinical use, but most are still in different phases of clinical trials. Key scientific milestones (**Figure 1**) have shaped the field. RSV was first identified in chimpanzees (1955) (5) and later in humans (1957) (6). A formalin-inactivated vaccine trial in 1966 failed due to vaccine-associated enhanced disease (7), highlighting the need for safer approaches. The approval of Palivizumab (Synagis) in 1998 marked the first licensed prophylactic intervention for severe lower respiratory tract disease caused by RSV (8, 9). Subsequent structural biology breakthroughs, including resolution of the post-fusion (2011) (10) and prefusion (2013) (11–13) F protein conformations, provided a molecular blueprint for rational vaccine design. In 2017, WHO designated RSV vaccine development a global priority (14), followed by resolution of the RSV polymerase complex (2019) (15) and identification of several host molecules proposed as viral entry receptors (16), such as CX3CR1 (17, 18), EGFR (19), heparan sulfate proteoglycans (HSPGs) (20), nucleolin (21), and IGF1R (22). Remarkable progress in both monoclonal antibody development and vaccine design has been driven by these advances, which enabled detailed characterization of RSV’s key antigenic target, the fusion (F) protein (10–13, 23–27), the focus of all currently available prophylactic options. Meanwhile, small-molecule anti-virals that target F or other RSV components have emerged as promising therapeutics (28–31). In this review, we will focus solely on prophylactics.

Among currently available prophylactic options, Palivizumab (8), a humanized monoclonal antibody targeting the RSV fusion (F) protein site II epitope accessible on both prefusion (pre-F) and postfusion (post-F) conformations (13), has been used for over two decades but requires monthly dosing and is restricted to high-risk infants due to cost and logistics. Nirsevimab is a next-generation long-acting antibody approved by the FDA for use in infants. It targets the pre-F site Ø (12), and provides season-long protection (32). Clesrovimab (Enflonsia), approved by the FDA in June 2025 (33), also targets the RSV F protein by binding to the site IV accessible on both pre-F and post-F (34), thereby broadening the available options for infant RSV prevention.

The pre-F conformation has also been shown to elicit potent neutralizing antibody responses, significantly accelerating progress in RSV vaccine design (11, 35, 36), leading to the licensure of the first RSV vaccines, Arexvy (GSK) and Abrysvo (Pfizer), for use in older adults and maternal immunization programs (37–39). The most recently approved vaccine, mRESVIA (Moderna), is an mRNA-based vaccine (38, 40). These approvals represent a historic milestone in RSV prevention and signal the transition from decades of experimental development to practical, population-level protection. Nevertheless, more work is needed to improve immunogenicity, long-lasting protection, and access for all high-risk groups, especially infants and young children.

Given these breakthroughs and ongoing challenges, a systematic overview of the current landscape of RSV vaccine research is both timely and necessary. This review focuses on RSV vaccine development, beginning with a brief overview of RSV molecular virology, reviewing monoclonal antibody prophylaxis, revisiting past vaccine failures, summarizing the current vaccine landscape, and highlighting key clinical trial outcomes to offer a comprehensive perspective. These insights, summarized here from original research, may be useful for informing the rational design of next-generation RSV vaccines, aiming to improve efficacy, enhance safety, and expand protection across high-risk populations.

## 2 Molecular virology of RSV, fusion protein, and antibody prophylaxis

### 2.1 RSV genome, proteins, and morphology

**Figure 2:**
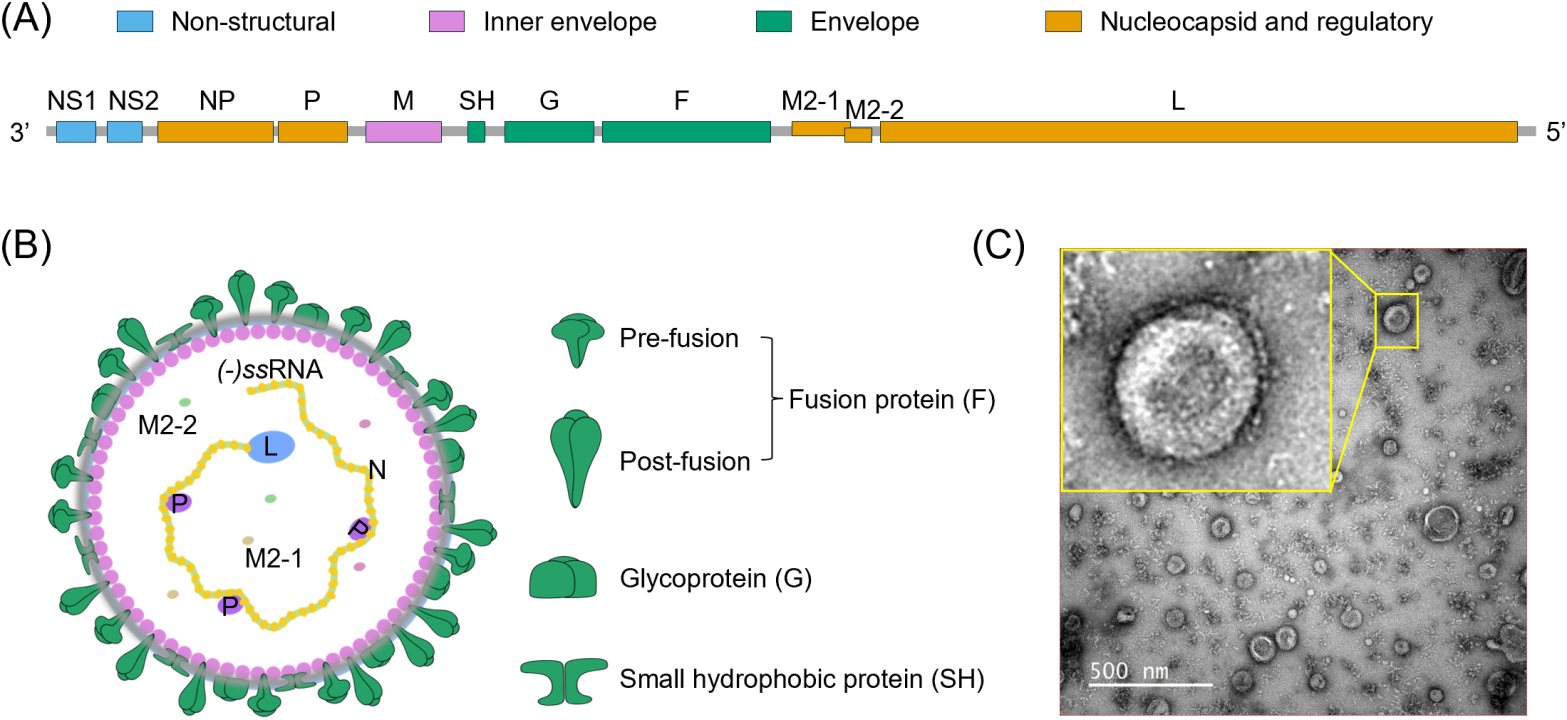
Genome organization, virion structure, and morphology of RSV. **(A)** Proportional schematic representation of the single-stranded negative-sense RNA genome of RSV. The genome contains 10 gene segments that can encode 11 distinct proteins classified into four functional categories. **(B)** Structural illustration of the spherical RSV virion. The viral surface contains three distinct structural proteins: the fusion glycoprotein (F), the attachment glycoprotein (G), and the small hydrophobic protein (SH). The fusion protein exists in two conformations: a pre-fusion state (thicker and shorter) and a post-fusion state (thinner and longer). The matrix protein (M) lies beneath the viral envelope, providing structural support. The viral genome, along with several nonstructural proteins and nucleocapsid-associated proteins, is located within the interior of the virion. **(C)** Negative-stain transmission electron microscopy (TEM) image of RSV particles in our hands. RSV was propagated in HEp-2 cells and harvested from the culture supernatant after 5 days post-infection. Viral particles were filtered through a 0.45-μm membrane prior to imaging under TEM. The virions are round with a diameter of ~150 nm and have many protrusions on the surface.

RSV is an enveloped, pleomorphic virus typically ~150 nm in diameter (**Figure 2**), that exhibits both spherical and filamentous forms, and belongs to the genus *Orthopneumovirus* in the family *Pneumoviridae* (41–44). Its genome is a linear, unsegmented, negative-sense, single-stranded RNA (-*ss*RNA) of approximately 15.2 kb, encoding 11 proteins, including 9 structural and 2 non-structural proteins (**Figure 2A**) (42, 45, 46). Among nine structural proteins, the outer viral membrane incorporates the fusion (F) protein, the attachment (G) protein, and the small hydrophobic transmembrane protein (SH) (**Figure 2B**). F mediates fusion between the viral and host cellular membranes, G facilitates viral attachment to the cell surface, and the function of SH remains unclear. Both F and G proteins have been shown to induce neutralizing antibodies and protective effects in animal models (47). Based on the reactivity of monoclonal antibodies to the F and G proteins, RSV is classified into subtypes A and B (41, 48, 49). The SH protein, due to its various post-translational modification forms, has an elusive function but is generally considered to play a role in immune evasion during the early stages of the RSV replication cycle (50). The matrix (M) protein, located on the inner side of the viral envelope, serves as a bridge connecting the viral membrane to the nucleocapsid and plays a critical role in the viral budding process (51). The nucleoprotein (N), phosphoprotein (P), and RNA polymerase (L) collectively form the ribonucleoprotein (RNP) complex, which encapsulates the viral genomic RNA and serves as a scaffold for replication and transcription mediated by the viral polymerase complex (52, 53). Viral genome transcription involves two additional proteins, M2–1 and M2-2. M2–1 acts as a transcription processivity factor, promoting RNA transcription, while M2–2 regulates the balance between RNA replication and transcription (54, 55). Near the 3’ promoter, RSV encodes two non-structural proteins, NS1 and NS2, which facilitate viral growth by modulating the activation and response of type I, II, and III interferon (IFN) (56–59).

### 2.2 RSV life cycle

During the infection process, RSV initially binds to receptors on the host cell membrane and enters the host cell via endocytosis or direct fusion at the cell surface (45). Following cellular entry, the virus releases the RNP complex, which initiates replication and transcription, producing the genomic and sub-genomic mRNAs required for viral replication. The genome is packaged into new RNP complexes, while sub-genomic mRNAs are translated into proteins on host endoplasmic reticulum ribosomes. These proteins are further processed in the Golgi apparatus before being transported, along with RNP complexes, to the plasma membrane, where viral assembly and budding occur and release the RSV virions, completing the replication cycle (43, 44, 60).

### 2.3 Pre-fusion F protein as a primary antigenic target

**Figure 3:**
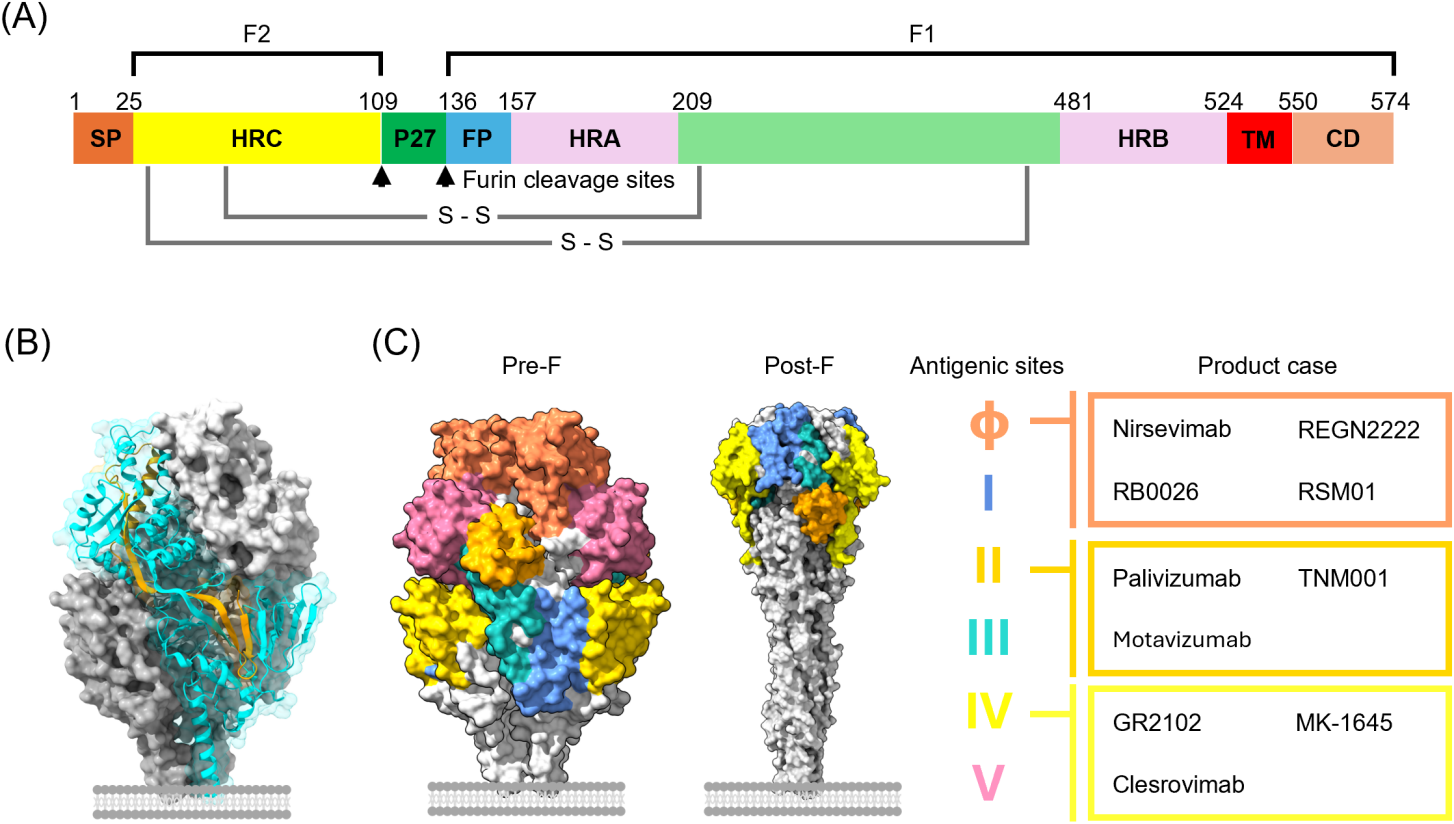
Schematic diagram of the RSV (A2 strain) fusion protein and the distribution of major antigenic sites. **(A)** Linear map of the RSV fusion glycoprotein (F). The immature precursor (F0) is proteolytically cleaved by host furin-like proteases at two multibasic cleavage sites, yielding the F2 (left) and F1 (right) subunits and releasing the intervening 27-residue peptide (pep27). Key structural elements are indicated, including the signal peptide (SP), fusion peptide (FP), heptad repeat A (HRA), heptad repeat B (HRB), heptad repeat C (HRC), transmembrane (TM), and cytoplasmic domain (CD). **(B)** Trimeric structure of the pre-fusion RSV F protein (PDB: 8YE3). Distinct protomers are shown in surface representation with different colors. One protomer is highlighted in cartoon format to illustrate the arrangement of F1 (Cyan) and F2 (Orange) subunits, which are covalently linked by conserved disulfide bonds (S–S) to form a stable heterodimer. Three such heterodimers assemble into the fusion-competent trimer that mediates viral entry. **(C)** Distribution of major antigenic sites on the pre-fusion (left, PDB: 8YE3) and post-fusion (right, PDB: 3RRR) conformations of the F protein. The accompanying table (right) lists representative monoclonal antibodies and vaccine candidates that target specific antigenic sites: Nirsevimab, RB0026 and RSM01 (site Ø); Palivizumab and Motavizumab (site II); and GR2102, MK-1645, and Clesrovimab (site IV).

A central determinant of RSV infectivity is the F protein, which is highly conserved and contains well-defined neutralizing epitopes (13). The F protein is synthesized as a precursor (F0) that is cleaved into two subunits, F1 and F2, linked by disulfide bonds (**Figure 3A**). F1 contains the fusion peptide, heptad repeat regions, and transmembrane domain, all critical for mediating membrane fusion, while F2 contributes to surface stabilization (**Figure 3B**) (61). Multiple potent neutralizing epitopes are located on the F protein, including antigenic sites Ø and V in the pre-F conformation, as well as sites I, II, III, and IV, which are present in both pre-F and post-F forms (**Figure 3C**) (12, 62–64).

**Figure 4:**
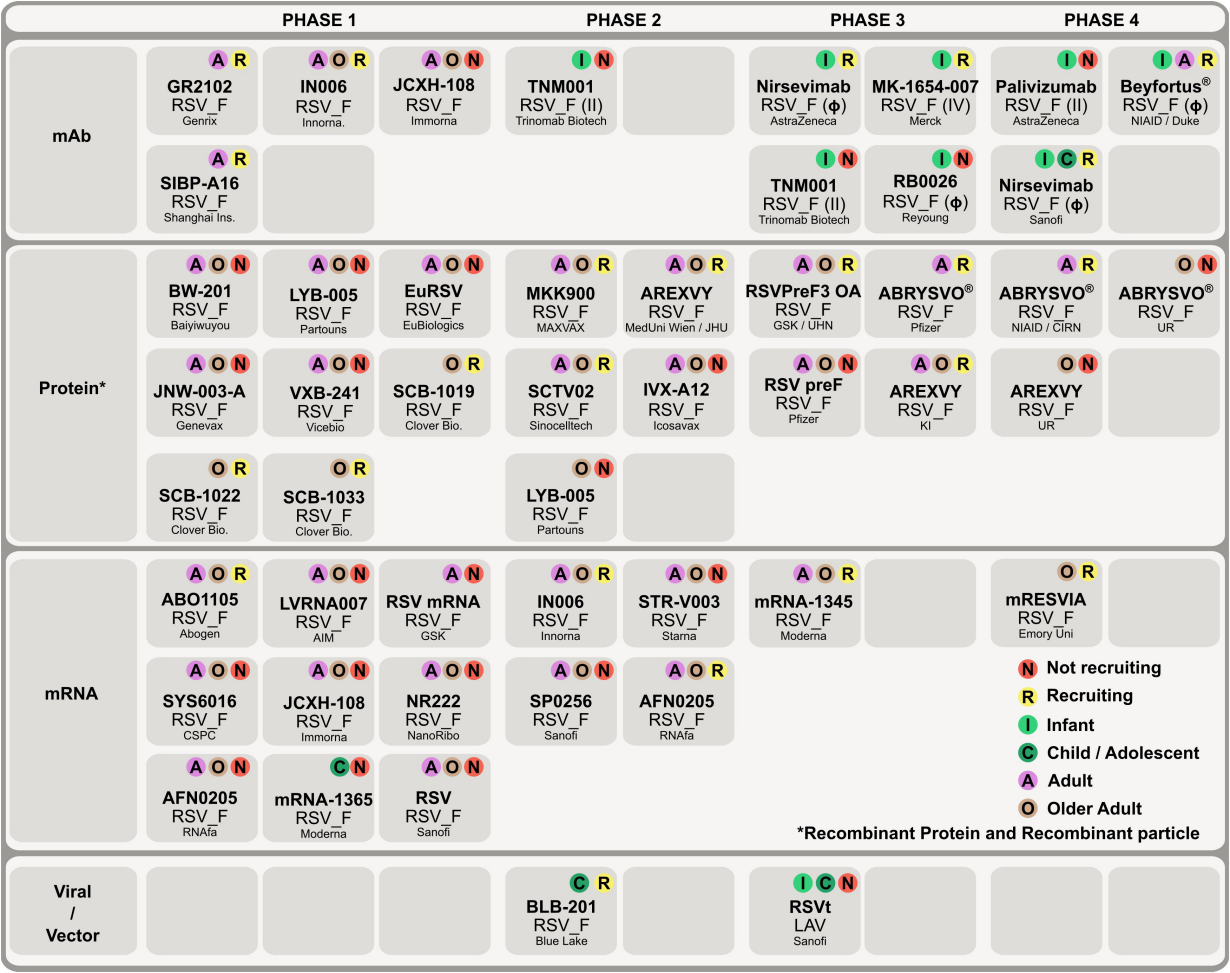
Snapshot of RSV monoclonal antibodies and vaccines under clinical evaluation. Currently approved and ongoing clinical trials for RSV vaccines and antibodies predominantly target the F protein. Prophylactic monoclonal antibody trials are concentrated in Phases 3 and 4, with enrollment primarily in infants. In vaccines, protein-based subunit candidates remain the most common across phases, while mRNA vaccines are emerging and largely in Phases 1-2. Most vaccine trials enroll adults and older adults, with viral-vector programs the main exception. Data source: PATH (77).

**Figure 5:**
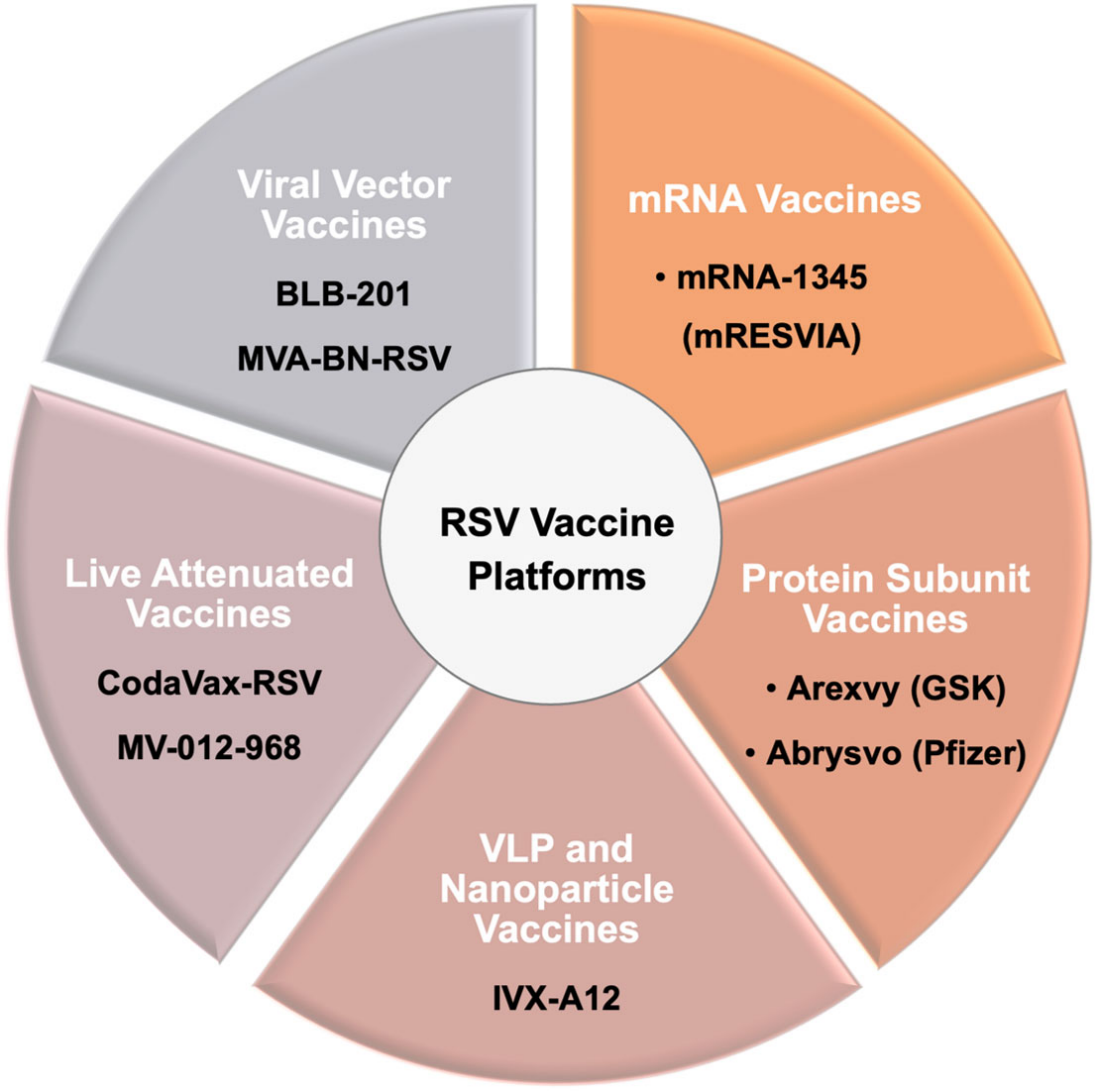
RSV vaccine platforms. Schematic representation of the major RSV vaccine platforms exemplified by some currently in clinical development or recently licensed. Protein subunit vaccines: Arexvy (GSK) and Abrysvo (Pfizer), both based on stabilized prefusion F protein antigens and approved by the U.S. FDA in 2023 as the first licensed RSV vaccines for older adults. mRNA vaccines: exemplified by mRNA-1345 (mRESVIA, Moderna), which was approved in 2024. Virus-like particle (VLP) and nanoparticle vaccines: IVX-A12 and DPX-RSV. Live attenuated vaccines: CodaVax-RSV and MV-012-968. Viral vector vaccines: BLB-201 and MVA-BN-RSV. Together, these platforms represent diverse technological strategies aimed at overcoming historical failures and providing effective prophylaxis against RSV across different target populations.

In RSV-infected individuals, the serum neutralizing antibody titers induced by the F protein are significantly higher (> 30-fold) than those induced by the G proteins, making the F protein a primary target for vaccine development (64–66). The F protein undergoes major conformational transitions, shifting from a metastable pre-F state to a stable post-F state during cellular entry of RSV (12). Neutralizing antibodies that specifically recognize pre-F (antigenic sites Ø and V) account for the majority of serum neutralizing activity and exhibit far greater potency than antibodies targeting epitopes exposed on both pre-F and post-F (12, 67). Thus, vaccines designed to present the F protein in its pre-F conformation elicit robust neutralizing antibody responses, establishing pre-F as the most promising target antigen for effective RSV vaccine development (24–27, 36, 68–70). Thanks to these advances in molecular understanding of RSV, several preventive options for RSV infections have been approved, including F-directed monoclonal antibody prophylaxis with Palivizumab, Nirsevimab, and, most recently, Clesrovimab (Enflonsia), as well as newly licensed vaccines, Arexvy (GSK), Abrysvo (Pfizer), and mRESVIA (Moderna). Meanwhile, multiple vaccine and antibody-based candidates remain under clinical investigation, many of which target the pre-F protein, as illustrated in **Figures 4**, **5** and discussed in the following sections.

### 2.4 F-directed monoclonal antibody prophylaxis

Historically, RSV-IVIG, the first polyclonal antibody preparation approved for prevention of severe RSV disease, was derived from pooled plasma from healthy blood donors and contained high titers of RSV-neutralizing antibodies (71, 72). Administration required monthly intravenous infusions by trained healthcare professionals, and its use was limited by high cost, logistical burden, variable efficacy, and batch-to-batch variability. Consequently, RSV-IVIG was withdrawn following the introduction of Palivizumab (73).

Palivizumab, derived from the parental monoclonal antibody mAb1129, is a humanized monoclonal antibody targeting antigenic site II of the F protein (8, 74). By binding to the F protein, it inhibits the fusion of the virus with host cell membranes, thereby preventing further infection. Palivizumab is primarily used for immunoprophylaxis in high-risk premature infants, but its therapeutic efficacy after infection is limited (75). Its high cost and requirement for monthly administration during the RSV season have constrained widespread use. According to the American Academy of Pediatrics (AAP), Palivizumab will be discontinued as of December 31, 2025 (76).

Long-acting monoclonal antibody prophylaxis has emerged as one of the major focuses for RSV prevention research, with two approved and several candidates undergoing clinical evaluation (77) (**Figure 4**). Nirsevimab-alip (Beyfortus), containing the active ingredient Nirsevimab, was approved by the FDA in July 2023 and recommended by AAP for the prevention of RSV infection in infants under one year of age (78). Unlike Palivizumab, Nirsevimab targets antigenic site Ø of the RSV pre-F protein and provides protection for up to five months (32, 79, 80). Its site of action is relatively conserved, and Nirsevimab escape variants are rare and have not increased over time, indicating its broad applicability (81). Enflonsia (Clesrovimab-cfor), developed by Merck, is another long-acting monoclonal antibody approved by the FDA in June 2025 (33), and it has been endorsed by the CDC as a preventive treatment for RSV infection in infants. Enflonsia is the first and only RSV prophylactic antibody administered at a uniform dose regardless of infant weight, significantly reducing the workload for healthcare providers while ensuring dosing safety (82). Enflonsia targets antigenic site IV accessible on both pre-F and post-F (34, 83).

## 3 RSV vaccine developments and platforms

In this section, we categorize RSV vaccine platforms and highlight key successes, including FDA-approved vaccines, while reviewing ongoing candidates under clinical evaluation (**Figure 4**). Platforms covered include mRNA-based, F protein subunit, virus-like particle and nanoparticle, live-attenuated, and viral vector approaches (**Figure 5**). In this review, the term “vaccines” is used broadly to refer to both licensed or approved vaccines and candidates currently undergoing clinical evaluation.

*History.* The development of RSV vaccines began in the 1960s, following the discovery of the virus. Inspired by the success of several formalin-inactivated vaccines at the time, researchers conducted clinical studies to evaluate the efficacy of a formalin-inactivated RSV vaccine (FI-RSV) adjuvanted with alum. Clear evidence indicates that F in the FI-RSV formula adopts the postfusion conformation, which induces abundant non-neutralizing antibodies and a lack of neutralizing antibodies (84), explaining why no neutralizing activity was previously observed (85). Regarding clinical results, many vaccinated infants who were subsequently exposed to natural RSV infection developed enhanced respiratory disease (ERD), with 80% of vaccine recipients requiring hospitalization and two fatalities reported (7). This was not observed in older children naturally infected with RSV. A widely accepted explanation for ERD is that it resulted from an imbalanced immune response to the RSV G and F proteins (86). The lack of pre-F and the dominance of G protein in the immune response to FI-RSV led to the development of immune complications in lung tissues, an imbalanced Th2 immune response, increased pulmonary eosinophilia, and ultimately bronchoconstriction in affected patients (87).

*Current rollouts.* After decades of research, RSV vaccine development has finally reached a major milestone with three FDA-approved vaccines for adults (88, 89): Arexvy (GSK) (38, 90), mRESVIA (Moderna) (91, 92), and Abrysvo (Pfizer) (37, 93). All three vaccines target the F protein. Arexvy and Abrysvo are subunit vaccines that utilize a stabilized pre-F protein as the immunogen (11). In contrast, mRESVIA is a novel mRNA vaccine that induces intracellular production of the pre-F protein, thereby eliciting a host immune response (91). These approved RSV vaccines represent a historic turning point and will be introduced in detail in their respective vaccine categories. The new adenovirus vector vaccine (Ad26.RSV.preF) has attracted considerable attention, in part due to its promising fold rise (GMFR, geometric mean fold rise) of neutralizing antibody titers (94) (**Table 1**).

**Table 1:** GMFR of serum neutralizing antibodies in selected vaccine trial participants.

Vaccine (sponsor)	Phase/Population	GMFR (95% CI)		Timepoint	References
		RSV-A	RSV-B		
mRESVIA (Moderna)	2/3***	8.0 – 8.9	4.9 – 5.5	Day 29	(92)
Arexvy (GSK)	3***	9.4 – 11.3	8.0 – 9.0	Day 30	(90)
Abrysvo (Pfizer)	3***	10.5 – 12.7	11.5 – 14.0	Day 30	(93)
Ad26.RSV.preF (Janssen)	2b***	12.1	9.4	Day 15	(94, 95)
	3**	10.5 – 14.3	n/a	Day 15	(96)
DS-Cav1(NIAID/NIH)	1**	7.5 – 12.6	6.4 – 9.8	Day 28	(97)
IVX-A12 (AstraZeneca)	1***	2.0 – 4.0	1.0 – 3.0	Day 28	(98)
ChAd155-RSV (GSK)	1/2*	4.2 – 8.1	n/a	Day 31	(99)

Vaccine trial participant population: *Children (12–23 months), **Adults (aged 18–59 years), *** Old adults (aged 60 years and above). n/a: not applicable.

### 3.1 mRNA-based vaccines

mRNA vaccines deliver RNA sequences encoding antigenic proteins into host cells via lipid nanoparticles (LNPs) or other carriers. Once internalized, the mRNA is translated into antigenic proteins, which induce antibody responses. These vaccines offer several advantages, including flexible antigen design, high safety, rapid development cycles, and scalability. However, a major limitation remains the thermal instability of mRNA, which poses challenges for degradation prevention and long-term storage (100).

*Early candidates.* mRNA-1777 (V171), jointly developed by Moderna and Merck, was the first mRNA vaccine designed to prevent RSV infection by encoding the pre-F to stimulate neutralizing activities (101). Phase 1 clinical trial results showed good tolerability across all dosage levels, with no serious adverse events reported (102). Immunologically, the vaccine significantly increased RSV-neutralizing antibody titers (2.5 – 3.9-fold) and enhanced CD4+ T-cell responses specific to the RSV pre-F protein. The later-optimized version, V172, introduced an additional interprotomeric disulfide bond in the F protein to further stabilize its pre-F state (69), which elicited a stronger antibody response than V171. However, both were suspended after Phase 1 trials due to shifts in the company’s strategic plans and the likely occurrence of severe side effects.

*mRESVIA (Moderna).* mRNA-1345 (mRESVIA) is the most clinically advanced mRNA-based RSV vaccine and a rationally optimized successor to the cytoplasmic tail deleted version of mRNA-1172, featuring multiple additional sequence modifications, resulting in pre-F antigen presentation (103). It encodes a stabilized pre-F protein, with modifications that enhance expression, folding, and stability, and uses LNP delivery to facilitate efficient cellular uptake and antigen presentation. Important modifications along with amino acid substitutions in the F protein include cavity-filling (V207L and S190F), interprotomer disulfide bonds (S155C and S290C), interprotomer disulfide bonds (A149C and Y458C), subunit linker (T103-GS-G145), and truncation at residue Y559 (103). This design improves both humoral and cellular immunogenicity compared with earlier constructs. mRESVIA became the first FDA-approved mRNA vaccine for RSV prevention in adults aged 18 years and older (40). Ongoing global clinical trials are investigating its use in broader populations, including pediatric cohorts, pregnant individuals for maternal immunization, and immunocompromised adults, as well as its co-administration with seasonal influenza vaccines. Published clinical data indicate that a single dose administered to healthy adults aged 65–79 induces high levels of neutralizing antibodies, with immune protection (more than 80%) lasting over 12 months (91, 92) (**Table 1**). Booster doses have also demonstrated strong immunogenicity (104). Post-vaccination reactions were generally mild to moderate, including injection site pain, fatigue, headache, and muscle soreness, with no reports of serious safety concerns or vaccine-associated enhanced disease (105). However, in clinical trials involving infants, the proportion of infants receiving Moderna’s RSV-mRNA vaccine candidate who developed severe or very severe LRTIs was significantly higher than that in the placebo group. The FDA announced the suspension of all RSV mRNA vaccine clinical trials in infants and RSV-naïve children (106). Collectively, mRESVIA provides a durable, well-tolerated, and scalable immunization option, marking a significant step toward integrating mRNA vaccine technology into RSV prevention programs beyond COVID-19.

### 3.2 Protein subunit vaccine

Protein subunit vaccines use highly antigenic protein fragments, either whole or partial, derived from pathogens to elicit protective immune responses without introducing the entire pathogen. They are considered safe due to their defined composition and lack of replicative capacity. The integration of structural biology with protein engineering has further expanded the potential applications of subunit vaccines. Currently, two subunit vaccines have been approved for the prevention of RSV-induced respiratory diseases, with several others in various clinical stages.

*DS-Cav1*. DS-Cav1 (VRC-RSVRGP084-00-VP) is a trimeric pre-F ectodomain stabilized with a foldon domain and four internal mutations (4.1DHFR_RSVAF) (70, 107). In its Phase 1 clinical trial, a single dose induced neutralizing antibody responses against both RSV-A and RSV-B subtypes (**Table 1**), with effects lasting for up to 44 weeks post-vaccination (97). This candidate could induce antibodies targeting all six antigenic sites on the F protein. Maternal immunization with the DS-Cav1 reported of neutralizing antibodies being transferred across the placenta, potentially extending the duration of protection in newborns (108). The core design was patented by NIH/NIAID and subsequently licensed to commercial partners, including GSK and Pfizer, for further development. Building on the pre-F stabilization strategy used in DS-Cav1, GSK and Pfizer later developed their respective commercial vaccines, Arexvy and Abrysvo.

*Arexvy (GSK).* Arexvy is the first RSV vaccine approved by the U.S. FDA and represents a landmark in RSV prevention. It is formulated as a single-dose subunit vaccine containing a stabilized pre-F of the RSV-A (RSVPreF3) combined with GSK’s proprietary adjuvant system AS01_E_ (109, 110). The active protein ingredient in Arexvy, RSVPreF3, is derived from and highly similar to the DS-Cav1 prototype. Both DS-Cav1 and the antigen used in GSK’s Arexvy are engineered versions of the ectodomain of the RSV-A strain A2 F protein designed to stabilize the protein in its prefusion conformation. Both incorporate the key DS and cavity-filling stabilizing concepts originally described for DS-Cav1. Arexvy is indicated for the prevention of RSV-mediated LRTI in individuals aged 60 years and older. The results from the Phase 3 trial demonstrated approximately a 10-fold rise in antibody titers against RSV-A and RSV-B (90) within one month of administration (**Table 1**). While these immunological markers declined at six months, protective efficacy remained clinically substantial, thus meaningful (111).

*Abrysvo (Pfizer).* Abrysvo (RSVpreF) is the only FDA-approved subunit vaccine indicated for both pregnant women (between 32 and 36 weeks of gestation) and adults aged 18 years and older (112). As with Arexvy, the stabilizing strategy used for the F protein is derived from DS-Cav1. It is a bivalent protein subunit containing stabilized pre-F proteins from both RSV-A and RSV-B. Notably, it does not require any immunostimulatory adjuvants, implying that the design of a stabilized prefusion F protein is key to protection rather than adjuvants. Abrysvo is primarily used to prevent RSV-mediated LRTI. Phase 3 clinical trials reported the high efficacy in preventing RSV-related LRTI, with protection rates exceeding 86% in individuals aged 60 and above (113) and, on average, a more than 10-fold rise in antibody titers (93) (**Table 1**).

The FDA has initiated post-marketing surveillance studies to assess the risk of Guillain-Barré Syndrome (GBS), a rare autoimmune disorder in which the immune system attacks peripheral nerves, potentially leading to muscle weakness or paralysis, following administration of these two vaccines, Arexvy (GSK) and Abrysvo (Pfizer). While preliminary evidence suggests a possible increase in GBS risk, current data are insufficient to establish a causal relationship (114). The investigation remains ongoing, and the FDA has stated that the benefits of vaccination with Arexvy and Abrysvo significantly outweigh the potential risks.

Unlike F-based vaccines, BARS13 targets the G protein and is co-administered with cyclosporine A, which serves as an immunomodulator and a diluent for reconstituted RSV-G (115). This combination is designed to stimulate regulatory T cells (Tregs), thereby mitigating the risk of vaccine-enhanced disease (VED) and significantly reducing its occurrence. Phase 2 clinical trial results demonstrated that BARS13 was well tolerated across all dosage groups, with no major safety concerns reported (116). The antibody response was dose-dependent, suggesting that optimal immunogenicity may require careful dose selection.

### 3.3 Virus-like particle and nanoparticle vaccine

Virus-like particle (VLP) vaccines consist of multimeric, self-assembling particles that mimic the size and structure of native viral particles but lack infectious genetic material. By displaying one or more viral antigens, often in a highly repetitive and multivalent fashion. VLPs effectively stimulate both humoral and cellular immunity while maintaining an excellent safety profile. Nanoparticle-based vaccines follow a similar principle, using engineered scaffolds to enhance antigen presentation and immune activation.

IVX-A12 is a bivalent VLP-based vaccine targeting both RSV and human metapneumovirus (hMPV), containing pre-F from each virus presented on VLPs. In clinical studies, IVX-A12 induced robust immune responses against both RSV (~ 1–4 fold increase) (**Table 1**) and hMPV (~ 1–5 fold increase) one month after vaccination, with no evidence of immune interference between the two antigens (98).

V-306 is a synthetic sVLP vaccine that presents a stabilized mimetic of antigenic site II (FSII, the Palivizumab binding site) of the F protein. The antigenic epitope, a protein mimetic (FSIIm), was stabilized through sequence modifications and disulfide bonds (117–119). The mimetic peptide is conjugated to a synthetic lipopeptide containing CD4^+^ T-helper epitopes. In Phase 1 clinical trials, V-306 elicited robust anti-FSIIm immune responses, though the increase in RSV-neutralizing titers was modest (118), Of note, no vaccine-related serious adverse events (SAEs) were reported, and there was no evidence of vaccine-associated enhanced respiratory disease (VAERD) (118). The safety does not seem to be a concern.

DPX-RSV targets the extracellular domain of the RSV-A SH protein, encapsulated using the DepoVax (DPX) oil-based delivery system (120). This formulation allows for slow antigen release, thus promoting active antigen uptake by immune cells and presentation to T cells, while reducing the risk of type III hypersensitivity reactions. In Phase 1 clinical trial, DPX-RSV was well-tolerated and elicited sustained SH-specific antibody production for up to 180 days after vaccination (121). Phase 1 of DPX-RSV has been completed without safety concerns. According to the company’s 2025 corporate review (Biovaxys), further clinical development of DPX-RSV is currently at the partner-seeking stage (122).

VLP- and nanoparticle-based RSV vaccines offer a highly versatile and safe platform for antigen presentation by mimicking native viral architecture without the risk of viral replication. Clinical data demonstrate their ability to induce robust and antigen-specific immune responses, including multivalent protection (IVX-A12) and epitope-focused immunity (V-306), while maintaining a favorable safety profile with no evidence of VAERD. However, the level of protection and neutralizing potency can vary depending on the antigen selected and the presentation strategy. Overall, these studies highlight the promise of VLP and nanoparticle vaccines as flexible, modular approaches for RSV immunization, with continued optimization still needed.

### 3.4 Live-attenuated vaccine

Live attenuated vaccines are developed by weakening the virulence of pathogens through laboratory techniques, allowing them to replicate in the host to a limited extent without causing severe disease. Most live-attenuated candidates are delivered intranasally to elicit strong humoral and cellular immune responses, similar to those observed during natural infection, with long-lasting immunity.

CodaVax-RSV is a genetically modified live attenuated candidate with reduced replication capacity and enhanced genetic stability. Its codon optimization design prevents reversion to the wild-type virus, ensuring safety. Phase 1 study achieved its primary safety endpoint and elicited strong anti-RSV specific cellular immune responses in elderly adults (123).

RSV-ΔG vaccine (ITV-RSV-ΔG) is generated by deleting the RSV G protein gene using reverse genetics (124). It is produced in Vero cells that stably express the G protein, resulting in viral particles that carry the G protein on their surface but lack the gene itself. Intranasal administration in cotton rats demonstrated immunogenicity and safety, though Phase 1 human trials did not show significant immune activation or increased neutralizing antibody levels post-vaccination (125).

MV-012–968 is engineered through codon deoptimization of the NS1, NS2, and G genes and deletion of the SH gene (115). These modifications reduce translation efficiency and viral replication. The vaccine strain was rescued in BSR-T7/5 cells and propagated in HEp-2 cells. Phase 1 trials confirmed its ability to induce RSV-specific mucosal immunity and demonstrated good tolerability in pediatric populations (126).

RSVΔNS2/Δ1313/I1314L (RSVt) contains deletions in the NS2 gene (an interferon antagonist) and codon modifications at positions 1313 and 1314 of the L gene (127). These changes render the virus replication-deficient at elevated temperatures (38–39 °C), reducing pathogenicity while preserving immunogenicity (127). Like MV-012-968, the virus was rescued in BSR-T7/5 cells and propagated in HEp-2 cells. Phase 1 trials showed good tolerability, with some participants experiencing mild upper respiratory symptoms (e.g., fever, rhinitis), but no serious adverse events were reported. The vaccine was later redesigned to enhance the proportion of pre-F in viral particles by introducing four amino acid mutations (I79M, K191R, T357K, N371Y), resulting in a more stable strain named ΔNS2-L19F-4M (128, 129). This improved stability offers advantages in manufacturing, storage, and distribution, ultimately reducing costs and improving accessibility. At the time of writing, and to the best of our knowledge, no specific or finalized clinical trial registration number is publicly available. The program is therefore presumed to be in a preclinical or transitional development phase.

RSV LID/ΔM2-2/1030s carries the deletion of the M2–2 protein to suppress viral replication and enhance antigen expression. It also incorporates a temperature-sensitive mutation (1030s) in the polymerase protein L to further restrict replication at elevated temperatures, improving safety. Phase 1 trials conducted in RSV-seronegative children demonstrated good safety and stability, with significant increases (> 4-fold) in serum neutralizing antibody levels and the development of immunological memory (130).

Live-attenuated RSV vaccines most closely recapitulate the immunological features of natural infection by efficiently infecting the upper respiratory tract and inducing robust mucosal and neutralizing antibody responses. Rational attenuation strategies, such as genetic recoding (e.g., CodaVax-RSV), targeted gene deletions, and temperature-sensitive mutations (e.g., ΔNS2-L19F-4M), have substantially improved safety profiles, although the risk of genetic reversion requires continued monitoring. Future live-attenuated vaccine designs could prioritize enhanced presentation of prefusion F to maximize immunogenicity. At the current stage, most candidates remain in preclinical development. The combination of genetic stability, strong immunogenicity, and intranasal delivery positions live-attenuated vaccines as a highly promising RSV vaccine platform.

### 3.5 Viral vector-based vaccines

Viral vector vaccines utilize genetically engineered, replication-deficient or non-pathogenic viruses as delivery vehicles to transport genes encoding target antigens into host cells, thereby inducing antigen expression and activating immune responses. Common viral vectors include adenovirus, measles virus, vesicular stomatitis virus (VSV), and modified vaccinia Ankara (MVA) (131).

BLB-201 is a chimeric viral vector vaccine based on parainfluenza virus type 5 (PIV5), engineered to express the full-length RSV F protein. Administered via intranasal spray, this vaccine significantly reduced patient stress. Phase 1 clinical trial demonstrated activation of humoral (≥ 1.5-fold rise), cellular, and mucosal immune responses, effectively lowering the risk of RSV infection (132). The vaccine was well tolerated, with no vaccine-related adverse events or serious medical incidents reported. BLB-201 has received fast track designation from the U.S. FDA and is currently undergoing phase 1/2a clinical trials (ClinicalTrials.gov ID: NCT05655182) in toddlers.

MVA-BN-RSV is a viral vector vaccine based on the MVA-BN platform (Modified Vaccinia Ankara) (133). Although capable of initiating viral protein expression in mammalian cells, MVA-BN does not replicate in most mammalian cells. This multi-antigen vaccine encodes five RSV proteins, F, G, SH, N, and M2-1, to induce broad neutralizing antibody responses and reduce antigen escape. In Phase 1 trials, MVA-BN-RSV elicited cellular immune responses to all five RSV proteins and humoral responses to both RSV subtypes (134). In Phase 3 clinical trials (133), this vaccine induced, on average, less than a 2-fold increase in neutralizing antibody titers against RSV A and B. While the protection level is modest, this vaccine showed acceptable reactogenicity with no major safety signals (133). These findings remain informative for the early development of MVA-BN–RSV vaccine.

PanAd3-RSV and MVA-RSV are replication-deficient viral vector vaccines using chimpanzee adenovirus and modified vaccinia Ankara, respectively. Both encode RSV F, N, and M2–1 proteins to stimulate humoral and cellular immunity (135). Animal studies showed effective protection against RSV via intranasal or intramuscular administration, with intranasal delivery inducing mucosal IgA responses against the F protein. Phase 1 clinical trials in healthy adults reported a ~2-fold increase in neutralizing antibodies and T-cell responses, and the result also proved that the combined use of PanAd3-RSV and MVA-RSV is safe and well tolerated in adults (136).

Ad26.RSV.preF vaccine uses replication-deficient adenovirus serotype 26 (Ad26) to deliver a gene encoding a structurally stabilized RSV-A2 pre-F protein (137). The low prevalence of pre-existing immunity to Ad26 in humans reduces the risk of vaccine failure. Its preclinical and clinical evaluations based on the mix of Ad26.RSV.preF and RSV pre-F protein showed promising efficacy and protection (94, 138). This vaccine demonstrated superior ability to induce durable RSV-specific humoral and cellular immunity lasting over two years in preclinical data (138). In the Phase 2b clinical trial (94), the study demonstrated on average more than a 10-fold increase in antibody titers (**Table 1**) at a level comparable to those of FDA-approved RSV vaccines (90, 92, 93). Ad26.RSV.preF also demonstrated protection (nearly 80%) that was maintained over multiple RSV seasons (95). The Phase 3 clinical trials in adults aged 18–59 years have recently been completed and showed age-dependent noninferior efficacy and protection, with acceptable safety (96). No further information is provided regarding the status of this vaccine afterward.

ChAd155-RSV is a viral vector vaccine using chimpanzee-derived adenovirus 155 (ChAd155) to encode RSV F, N, and M2–1 proteins. Phase 1 clinical trial results in adults showed that ChAd155-RSV increased specific humoral (~ 2.5-fold rise) and cellular immune responses without causing significant safety concerns (139). While the vaccine induced specific humoral (more than 4-fold rise) (**Table 1**) and cellular immune responses across age groups and was well-tolerated in phase 1/2 clinical studies, its suboptimal protective efficacy (~ 27%) in young infants later halted its further development (99, 140).

rBCG-N-hRSV is a recombinant vaccine candidate based on attenuated Mycobacterium Bovis BCG, engineered to express the RSV N protein (141). The vaccine aims to induce immunity against both tuberculosis and RSV. rBCG-N-hRSV demonstrated high efficacy in mice and neonatal calves, inducing robust humoral and cellular immune responses. Phase 1 human trials further showed its safety and ability to elicit specific immune responses (141, 142).

SeVRSV is an RSV vaccine candidate based on Sendai virus (SeV), which expresses the RSV F protein. SeV, a murine parainfluenza virus type 1 (PIV-1), also offers potential protection against human PIV-1 (143, 144). The vaccine is designed to provide dual protection (145), but it did not elicit, which did not reach the expected levels of humoral and cellular immune responses in Phase 1 trials.

Among these vector-based vaccine candidates, BLB-201 remains under clinical evaluation, whereas several others, such as a small trial in Japan of Ad26.RSV.preF (146) and ChAd155-RSV (140) have been discontinued. According to company disclosures, some programs were terminated primarily for strategic considerations rather than clear safety concerns. Of note, Ad26.RSV.preF demonstrated sustained protective efficacy over multiple RSV seasons (94, 95), indicating that adenoviral vector immunity did not abrogate vaccine-induced protection. While adenoviral vectors elicit vector-specific immune responses, available evidence suggests that anti-Ad26 immunity does not necessarily impair immune responses to subsequent Ad26-based vaccinations with heterologous inserts. For multivalent constructs such as ChAd155-RSV, the simultaneous expression of multiple antigens may introduce additional challenges in optimizing the balance of antigen expression and immunogenicity. These observations suggest that discontinuation of vector-based RSV vaccines reflects strategic development decisions rather than fundamental limitations of the platform.

## 4 Conclusion

RSV prevention has entered a new era. Approvals of F protein-based vaccines (Arexvy, Abrysvo, and mRESVIA) and long-acting monoclonal antibodies (Nirsevimab and Clesrovimab) mark a decisive shift from decades of scientific discovery to meaningful clinical outcomes. Central to this progress has been the elucidation of the molecular and structural details of the prefusion F protein, which has advanced rational antigen design across diverse platforms, such as protein subunits, mRNA, viral vectors, and live-attenuated candidates. These approaches aim to elicit durable immunity while maintaining a favorable safety profile.

Despite significant progress made in preventing RSV infections, many challenges remain. These challenges include: (1) the genetic diversity of RSV subtypes and associated risks of immune evasion, (2) incomplete understanding of mucosal immunity against RSV, and (3) the high cost of vaccines.

Regarding RSV subtypes and their associated immune evasion, genetic variations, especially the resulting sequence changes in critical proteins during ongoing RSV evolution, could reduce the effectiveness of current vaccines and monoclonal antibodies. A notable, wide genomic characterization of patient samples collected by the Johns Hopkins Health System during the 2023–2024 RSV season identified amino acid substitutions in both the G and F proteins of circulating RSV strains, some of which may be related to antigenic epitopes or antibody escape mechanisms (147). Proactive global molecular surveillance of RSV is urgently needed, and WHO is actively advancing this effort by implementing an online platform, *RespiMart* (148). It is a central data platform for the exchange, coordination, integration, and storage of surveillance data on respiratory viruses with epidemic and pandemic potential, including influenza, SARS-CoV-2, and RSV. This platform facilitates cooperation and data sharing to guide the prevention of RSV and the development of potential anti-RSV therapeutics.

A limited understanding of mucosal immune responses to RSV continues to hinder the optimization of clinical outcomes. Key mechanisms that govern protective mucosal immunity, including the dynamics of secretory IgA, tissue-resident memory T cells, and local innate responses, remain poorly understood. The knowledge gaps may help explain the lack of licensed RSV vaccines or prophylactic antibodies administered via mucosal routes. Supporting this challenge, intranasal administration of an adeno-RSV vaccine in mice showed that only co-delivery of an RSV F-protein–expressing adenoviral vector (Ad-F) with the adjuvant IL-1β generated robust F-specific antibodies and enhanced viral clearance, whereas Ad-F alone failed to induce strong immune activation (149). Consistently, a recent retrospective review of mucosal-associated vaccines against respiratory infectious diseases suggests that mucosal immunization can achieve strong immunogenicity, safety, and protection, but typically when paired with tailored adjuvants or delivery vehicles (150). Thus, gaps in our understanding of mucosal immune correlates of protection continue to limit rational vaccine design and contribute to incomplete or short-lived immunity, highlighting the need for strategies capable of reliably inducing durable upper and lower airway mucosal responses. Such noninvasive approaches also hold practical advantages, as they could reduce patient vaccine hesitancy.

Affordability is always one of the most significant challenges. The complexity of research, production, storage, and distribution inevitably drives costs upward, posing barriers to equitable access and large-scale implementation. According to the U.S. Centers for Disease Control and Prevention (CDC) vaccine price report (2025), the average cost per RSV vaccine dose is approximately $300, even in high-income countries, raising concerns about affordability. A WHO-funded economic evaluation further suggested that seasonal administration of monoclonal antibodies or maternal vaccines could be more cost-effective than year-round vaccination (151) though this optimization strategy still requires additional real-world data. Therefore, efforts to improve thermostable formulations, simplify delivery routes, and reduce manufacturing costs will be crucial steps in translating scientific advances into sustainable global impact.

Overall, in a broad and generalizable sense, we believe that the key priorities for the field now focus less on proving that protection is possible and more on optimizing breadth, durability, and access. These include: (1) extending protection to infants through maternal immunization, optimized monoclonal antibody strategies, and next-generation pediatric vaccines; (2) improving durability of immunity, particularly in older adults, through better antigen design, boosting strategies, and formulation refinements; (3) developing next-generation vaccines, such as nasal spray (intranasal) vaccines, live-attenuated vaccines, and particle-based formulations that stimulate strong immune defenses in the nose and airways, where RSV first enters the body; and (4) ensuring global impact through thermostable formulations, simplified dosing, and accessible and affordable for people in low- and middle-income countries. Of note, strategies that protect infants through maternal vaccination remain very promising, but they require close monitoring for safety signals, such as preterm birth, and careful adjustment of immunization timing.

In conclusion, converging insights from molecular virology, structural biology, and preclinical and clinical studies have positioned the field to deliver RSV vaccines and antibody prophylaxis that are durable, scalable, and globally accessible. These advances are expected to fulfill the long-standing promise of F protein, focused strategies, and significantly reduce the burden of severe RSV disease across all high-risk populations worldwide.
